# Percent ash‐free dry weight as a robust method to estimate energy density across taxa

**DOI:** 10.1002/ece3.5775

**Published:** 2019-11-25

**Authors:** Jacob Weil, Marc Trudel, Strahan Tucker, Richard D. Brodeur, Francis Juanes

**Affiliations:** ^1^ Department of Biology University of Victoria Victoria BC Canada; ^2^ St. Andrews Biological Station Fisheries and Oceans Canada St. Andrews NB Canada; ^3^ Pacific Biological Station Fisheries and Oceans Canada Nanaimo BC Canada; ^4^ National Marine Fisheries Service Northwest Fisheries Science Center, NOAA Newport OR USA

**Keywords:** bioenergetics, diet analysis, dry weight, energy density, food webs, percent ash‐free dry weight, predictive model, water content

## Abstract

Determining how energy flows through ecosystems reveals underlying ecological patterns that drive processes such as growth and food web dynamics. Models that assess the transfer of energy from producers to consumers require information on the energy content or energy density (ED) of prey species. ED is most accurately measured through bomb calorimetry, but this method suffers from limitations of cost, time, and sample requirements that often make it unrealistic for many studies. Percent dry weight (DW) is typically used as a proxy for ED, but this measure includes an indigestible portion (e.g., bones, shell, salt) that can vary widely among organisms. Further, several distinct models exist for various taxonomic groups, yet none can accurately estimate invertebrate, vertebrate and plant ED with a single equation. Here, we present a novel method to estimate the ED of organisms using percent ash‐free dry weight (AFDW). Using data obtained from 11 studies diverse in geographic, temporal and taxonomic scope, AFDW, DW as well as percent protein and percent lipid were compared as predictors of ED. Linear models were produced on a logarithmic scale, including dummy variables for broad taxonomic groups. AFDW was the superior predictor of ED compared to DW, percent protein content and percent lipid content. Model selection revealed that using correction factors (dummy variables) for aquatic animals (AA) and terrestrial invertebrates (TI) produced the best‐supported model—log_10_(ED) = 1.07*log_10_(AFDW) − 0.80 (*R* ^2^ = 0.978, *p* < .00001)—with an intercept adjustment of 0.09 and 0.04 for AA and TI, respectively. All models including AFDW as a predictor had high predictive power (*R* ^2^ > 0.97), suggesting that AFDW can be used with high degrees of certainty to predict the ED of taxonomically diverse organisms. Our AFDW model will allow ED to be determined with minimal cost and time requirements and excludes ash‐weight from estimates of digestible mass. Its ease of use will allow for ED to be more readily and accurately determined for diverse taxa across different ecosystems.

## Graphical abstract

Determining how energy flows through ecosystems reveals underlying ecological patterns that drive processes such as growth and food web dynamics. Models that assess the transfer of energy from producers to consumers require information on the energy density (ED) of prey species, however, measuring this value carries limitations of cost, time, and sample requirements. Here, we present a novel method to accurately estimate the ED of invertebrates, vertebrates, and plants using a single equation, with minimal cost and time requirements using percent ash‐free dry weight.

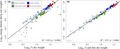

## 1 INTRODUCTION

Tracing energy flow through ecosystems has been used since Lindeman (1942) as a tool to answer questions about growth, species interactions, and community dynamics. An assessment of energy fluxes in ecosystems requires not only the quantification of the diet of consumers (Nielsen, Clare, Hayden, Brett, & Kratina, 2018), but also a determination of the energy density (ED) of both the prey and consumers (Cummins & Wuycheck, 1971). ED is a commonly used currency in ecology to ask questions related to growth, energy budgets, waste metabolism, and foraging behavior across a diversity of species (Chen, Thompson, & Dickman, 2004; Herrera, Osorio, & Mancina, 2011; Litzow, Piatt, Abookire, & Robards, 2004; Peckham et al., 2011; Rodgers & Sinclair, 1997). It is a key parameter in a wide variety of bioenergetics and growth models (e.g., Benoit‐Bird, 2004; Deslauriers, Heironimus, & Chipps, 2016; Maino & Kearney, 2015) but can be quite sensitive to error (Bartell, Breck, Gardner, & Brenkert, 1986). This error remains an important source of variability as somatic energy content and composition can vary substantially depending on spatial context (Dessier et al., 2018; Ruck, Steinberg, & Canuel, 2014; Schultz & Conover, 1997), season sampled (Chen et al., 2004; Pedersen & Hislop, 2001), or ontogenetic stage (Lawson et al., 2018; Woodland, Hall, & Calder, 1968). Thus, accurate measurements of ED are imperative to the validity of predictions made from these models.

Bomb calorimetry is the most direct approach for measuring ED, though it is also time‐consuming, costly and can have sample requirements that are challenging for estimating ED of smaller species (typically at least 25 mg of dry sample is required, Cummins & Wuycheck, 1971). When bomb calorimetry is not feasible, ED values are often borrowed from the literature or estimated using alternative methods. Proximate analysis makes use of conversion factors for major body constituents such as lipid and protein and converts them into gross energy content (e.g., Battam, Richardson, Watson, & Buttemer, 2010; Logerwell & Schaufler, 2005). While proximate analysis is regularly employed in lieu of direct measurement, it entails greater time and cost requirements than bomb calorimetry. In addition, lipid and protein extractions involve intricate methods than can introduce further error into estimates. There are also no standardized conversion factors for proximate constituents, even among a single class such as fishes (values can range from 17.2–23.9 kJ/g for protein and 34.7–39.8 kJ/g for lipid; Brett, 1995). As a result, ED estimates from proximate analysis can differ substantially from those obtained from bomb calorimetry (Craig, 1977).

Other inference methods have been developed based on observed relationships between organic components and ED. The most common alternative to infer ED for bioenergetics studies is through the relationship between energy content and percent dry weight (DW). In aquatic organisms, this relationship exists due to the negative association between percent fat and protein with water content (Craig, 1977; Flath & Diana, 1985). A predictive model to estimate ED based on these assumptions was first developed for fish by Hartman and Brandt (1995). Several such relationships have since been developed in the aquatic realm for individual species or locations (e.g., Ciancio, Pascual, & Beauchamp, 2007; Trudel, Tucker, Morris, Higgs, & Welch, 2005) as well as more generally for fish (Hartman & Brandt, 1995) and terrestrial and aquatic invertebrates (James et al., 2012). The most obvious advantage of employing these models is that they greatly reduce time and cost requirements, allowing researchers to obtain quick and easy estimates of ED for a large number of samples since all that is required is drying and weighing samples.

Models used to estimate ED from DW commonly assume that DW reflects the digestible energy or organic content of an organism and should thus be directly associated with ED. However, variability in inorganic material such as bone (Cameron, 1985), salt (Arai, 1997), or calcium carbonate shells (Lalli & Gilmer, 1989) between invertebrates and vertebrates as well as aquatic and terrestrial organisms may introduce error into models that predict ED, and may preclude the development of a general relationship to predict ED from DW across both aquatic and terrestrial organisms. This material typically comprises the indigestible ash‐weight of organisms. Ash‐weight can be measured following the determination of DW by burning off organic matter in a muffle furnace at high temperatures for several hours (Cummins & Wuycheck, 1971). This mass can then be subtracted from the DW fraction to give a measure of total organic content (Lucas, 1994) or percent ash‐free dry weight (AFDW). Hence, AFDW may provide a more accurate estimate of digestible energy across a wide range of taxa.

A second assumption made by models that estimate ED from DW is that a negative relationship exists between lipid and water content (Flath & Diana, 1985). While this may be true for animals, plants, and algae rely much more heavily on carbohydrates for storage as well as structure in their cell walls. As carbohydrates have a lower ED than high‐energy constituents like lipids, one would expect a lower estimate of ED in these organisms for the same value of AFDW. A novel model to estimate ED from AFDW would remove the error associated with variability in inorganic matter and provide a more accurate estimate of ED while remaining economical with respect to both time and cost. This model, however, would likely need to incorporate expected taxonomic differences between groups that vary greatly in proximate composition.

In this study, we investigated a novel, predictive model for estimating wet weight ED using percent AFDW across a wide range of aquatic and terrestrial organisms. Other common predictors, including percent dry weight, percent protein content, and percent lipid content were examined to compare their predictive power in estimating ED. We expected AFDW to be a superior predictor of ED relative to DW, percent protein content, and percent lipid content. Across taxonomic groups, we also expected to observe a lower value for ED in plants than for animals at the same value of DW or AFDW due to a higher reliance on low‐energy carbohydrates by algae and plants for storage and cell structure. A model that estimates wet weight ED from AFDW would allow for standardization among individuals with a variable component of inorganic matter that does not contribute to digestible energy for predators. AFDW also suffers less from time and cost restraints and allows for the accurate estimation of ED values for very small organisms.

## 2 MATERIALS & METHODS

### 2.1 Data collection

To determine the predictive power of AFDW in estimating the ED of both aquatic and terrestrial organisms, data were obtained from the literature that met the following selection criteria: ED was directly measured via bomb calorimetry, AFDW was measured and at least one other predictor variable was measured (either dry weight, percent protein content, or percent lipid content). Using the Web of Science database, the search terms “energy content”, “somatic energy”, “energy density”, “energetic value”, “caloric content”, “ash‐weight”, “dry weight”, and “ash‐free dry weight” were used to select studies that met our criteria. Originally, all taxonomic groups were to be included. However, only two papers were found that included data for AFDW and ED in terrestrial vertebrates (20 observations; Holmes, 1976; Myrcha & Pinowski, 1970) and these records represented very low taxonomic coverage (only passerine birds). As such, we excluded terrestrial vertebrates from our model development. Generally, papers that contained AFDW data also contained DW information, however, very few also listed percent protein and percent lipid content. Thus, data were subset into two groups that contained (a) only DW and AFDW measurements and (b) DW and AFDW measurements as well as percent protein and lipid content. The two datasets were analyzed separately.

### 2.2 Linear models

A series of linear models were developed for each of the two datasets. ED values (kJ/g wet weight) were plotted against predictor variables (DW, AFDW, percent protein, and percent lipid) for each subset. All the data were log_10_ transformed prior to performing the analyses. Dummy variables (a value of 0 or 1) were assigned to each data point corresponding to the broad taxonomic grouping of the organism. Broad taxonomic groups included aquatic invertebrates (AI), aquatic vertebrates (AV), terrestrial invertebrates (TI) and aquatic plants and algae (APA). Dataset two did not contain any TI data. APA organisms were used in the base model and given zeroes for all dummy variables. Two other dummy variable groups were included to determine if significant differences in model predictions exist between aquatic animals (AA) and terrestrial animals, and between animals (AN) and APA. Candidate models included a single continuous predictor (AFDW, DW, percent protein, or percent lipid) and a subset of nonoverlapping dummy variables (e.g., AA and AI would not be included in the same model), resulting in 22 candidate models for dataset one (for AFDW and DW) and 20 candidate models for dataset two (including all predictor variables). Candidate models were limited to one continuous predictor per model due to significant colinearity among predictors (variance inflation factor > 10).

Both model sets were compared independently using Akaike's Information Criterion corrected for small sample sizes (AICc; Burnham & Anderson, 2002). Candidate models were ranked based on their AICc scores and log‐likelihood (Log*L*), *R* ^2^ adjusted for number of predictors, cumulative Akaike weights (*w_i_*), and the difference between the given, and best‐fitting model (∆*_i_*) was calculated for each model (Burnham & Anderson, 2002). The best‐fitting model was determined by the lowest AICc score (a ∆*_i_* value of 0.0); however, a ∆*_i_* of <2 was also considered to have substantial support (Burnham & Anderson, 2002). Any model with a ∆*_i_* between 4 and 7 was considered to have considerably less support, and ∆*_i_* values >10 were assumed to have essentially no support. The *w_i_* score is considered analogous to the probability that a candidate model is the best supported of the given set of models. The standardization of continuous variables to a mean of zero and standard deviation of one is recommended when interpreting the effect of dummy variables (Legendre & Legendre, 1998). However, the continuous predictors were left unstandardized as no differences in results were observed after standardization. Leaving data in this format allowed for the simpler application of models in predicting ED. All statistical analyses were conducted using R statistical software (R Core Team, 2017).

### 2.3 Cross‐validation

To assess the error associated with the best supported models, we performed an iterative cross‐validation on our dataset. Data were randomly divided into either training (80% of observations) or testing datasets (20% of observations). The training dataset was used to develop a predictive equation based on each of the five best supported models determined through AICc. These equations were used to predict ED values for the testing dataset. Root mean square error (RMSE) was calculated to evaluate the discrepancy between predicted and observed values. This process was iterated 10,000 times for each of the five best supported models.

## 3 RESULTS

Eleven publications were found that met the criteria for dataset one (publications that measured both DW and AFDW), of which 200 organisms, ontogenetic stages or seasonal records were tabulated. Broad taxonomic coverage included 107 records of aquatic invertebrates, 30 of aquatic vertebrates, 43 of aquatic plants and algae, and 20 of terrestrial invertebrates. Spatial coverage included oceanic waters from the North and South Atlantic as well as the Pacific Ocean, freshwater data from Europe and terrestrial sources from North America and Australia. Terrestrial insects reared in the laboratory were also included as a source in the model (Woodland et al., 1968). The temporal extent of sampling occurred in every season across a five‐decade span (1962–2007). Three publications met our criteria for the direct measurement of ED and AFDW that also included measurements of percent protein and percent lipid content (dataset 2). These 26 organisms included 19 aquatic invertebrates, 2 aquatic plants, and 5 aquatic vertebrates and were sampled in the Atlantic and Pacific Oceans between 1962 and 2007.

**Figure 1:**
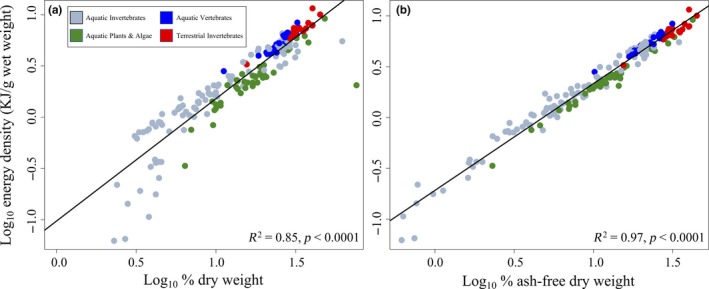
Model comparison for full dataset of literature values reporting percent dry weight (a) and percent ash‐free dry weight (b); both axes on a logarithmic scale

**Figure 2:**
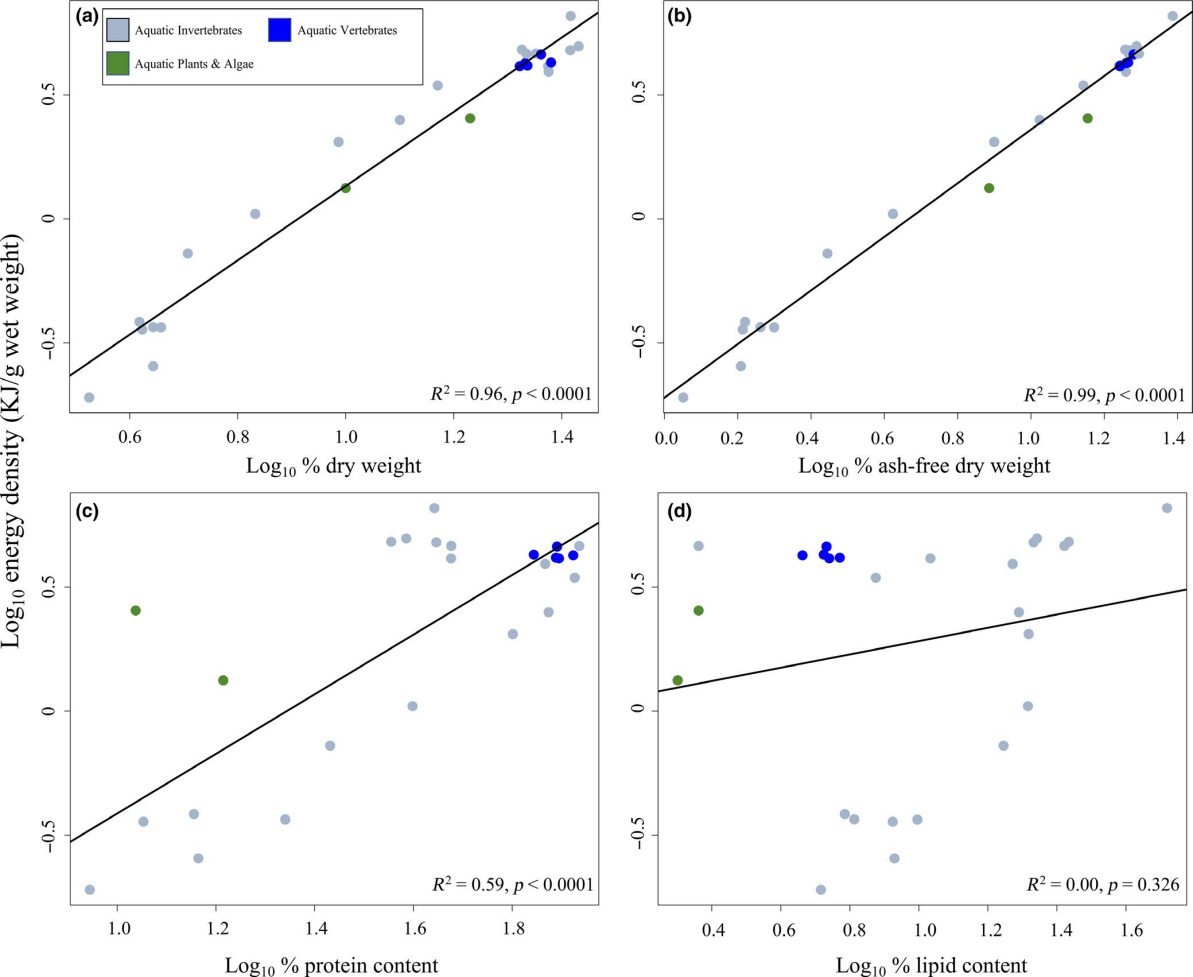
Model comparison for trimmed dataset including all hypothesized predictors of energy density: (a) log_10_ percent ash‐free dry weight, (b) log_10_ percent dry weight, (c) log_10_ percent protein content, (d) log_10_ percent lipid content

Using dataset one, a significant positive relationship was observed between ED and DW (Figure 1a, *R* ^2^ = 0.85 *p* < .0001). An even stronger positive relationship was observed between ED and AFDW (Figure 1b, *R* ^2^ = 0.97, *p* < .0001). Shifting from DW to AFDW predictors removed the bias in ED predictions for aquatic invertebrates at relatively low values of DW and AFDW. Similar, positive relationships emerged using dataset two. ED was again significantly, positively related to DW (Figure 2a, *R* ^2^ = 0.96, *p* < .0001). ED was also significantly, positively related to AFDW (Figure 2b, *R* ^2^ = 0.99, *p* < .0001). ED was significantly, positively related to percent protein; however, the strength of the relationship was much less than was observed for either DW or AFDW (Figure 2c, *R* ^2^ = 0.59, *p* < .0001). Surprisingly, ED showed no significant relationship to percent lipid (Figure 2d, *R* ^2^ = 0.00, *p* = .3).

AICc model selection results for dataset one revealed that all models that included AFDW as a predictor were superior to those including DW, with AIC differing by more than 310 between the best performing DW model relative to the worst performing AFDW model (Table 1). Of the 22 candidate models, the best‐supported model to predict ED was AFDW + aquatic animals (AA) + terrestrial invertebrates (TI; Table 1). A second model predicting ED by AFDW + aquatic invertebrates (AI) + aquatic vertebrates (AV) + TI also received substantial support, which splits AA into its two separate parameters (AI and AV). Three additional models received some support in the model selection. AFDW + AA, AFDW + AI + AV and AFDW + AN all exhibited nonzero *w_i_* scores during model selection (Table 1). The *R* ^2^ value for all five of the top models was 0.98. The predictive equations including correction factors for broad taxonomic groups in these top models are presented in Table 2.

**Table 1:** Log‐likelihood (Log*L*) and Akaike's information criterion corrected for small sample sizes (AICc) for generalized linear models relating energy density to percent dry weight (DW) and percent ash‐free dry weight (AFDW), including dummy variables (value of 0 or 1) for large taxonomic groups (AA, aquatic animals; AI, aquatic invertebrates; AN, animal; AV, aquatic vertebrates; TI, terrestrial invertebrates; aquatic plants and algae were used in the base model and therefore were not given a dummy value). The number of model parameters (*k*), cumulative Akaike weights (*w_i_*), adjusted percent *R* ^2^ values and the difference between the given and best‐fitting model (∆*_i_*) are presented for each candidate model

Model	*k*	Log*L*	AICc	∆*_i_*	*w_i_*	*R* ^2^
AFDW + AA + TI	5	273.03	−535.76	0.00	0.55	0.98
AFDW + AI + AV + TI	6	273.40	−534.37	1.39	0.27	0.98
AFDW + AA	4	270.28	−532.36	3.39	0.10	0.98
AFDW + AI + AV	5	270.42	−530.54	5.22	0.04	0.98
AFDW + AN	4	269.28	−530.36	5.39	0.04	0.98
AFDW + AI	4	255.32	−502.44	33.31	0.00	0.97
AFDW + AI + TI	5	255.33	−500.35	35.41	0.00	0.97
AFDW + AV	4	250.15	−492.09	43.67	0.00	0.97
AFDW + AV + TI	5	250.16	−490.02	45.74	0.00	0.97
AFDW	3	245.37	−484.62	51.14	0.00	0.97
AFDW + TI	4	245.92	−483.63	52.13	0.00	0.97
DW + AN	4	90.41	−172.61	363.15	0.00	0.87
DW + AI + AV + TI	6	91.79	−171.14	364.62	0.00	0.87
DW + AA + TI	5	90.59	−170.87	364.88	0.00	0.87
DW + AA	4	83.73	−159.26	376.50	0.00	0.86
DW + AI + AV	5	84.33	−158.35	377.41	0.00	0.86
DW + AV + TI	5	83.15	−155.99	379.76	0.00	0.86
DW + AV	4	80.20	−152.19	383.57	0.00	0.85
DW + AI + TI	5	80.98	−151.66	384.10	0.00	0.85
DW + AI	4	78.71	−149.22	386.53	0.00	0.85
DW + TI	4	78.64	−149.08	386.67	0.00	0.85
DW	3	77.26	−148.40	387.36	0.00	0.85

**Table 2:** Equations and correction factors (CF) for all generalized linear models relating energy density (kJ/g wet weight) to both percent dry weight (DW) and percent ash‐free dry weight (AFDW) including dummy variables (value of 0 or 1) for large taxonomic groups; AA, aquatic animals; AI, aquatic invertebrates; AN, animals; AV, aquatic vertebrates; TI, terrestrial invertebrates. The difference between the given and best‐fitting model (∆*_i_*) and adjusted percent *R* ^2^ values are presented for each candidate model

Model	∆*_i_*	Equation	CF	*R* ^2^
AFDW + AA + TI	0	log_10_(ED) = 1.07*log_10_(AFDW) − 0.80	AA = 0.09, TI = 0.04	0.98
AFDW + AA	3.39	log_10_(ED) = 1.08*log_10_(AFDW) − 0.79	AA = 0.07	0.98
AFDW + AN	5.39	log_10_(ED) = 1.05*log_10_(AFDW) − 0.78	AN = 0.08	0.98
AFDW + AI	33.31	log_10_(ED) = 1.08*log_10_(AFDW) − 0.77	AI = 0.05	0.97
AFDW + AI + TI	35.41	log_10_(ED) = 1.08*log_10_(AFDW) − 0.77	AI = 0.05, TI = 0.00	0.97
AFDW + AV	43.67	log_10_(ED) = 1.04*log_10_(AFDW) − 0.71	AV = 0.04	0.97
AFDW + AV + TI	45.74	log_10_(ED) = 1.04*log_10_(AFDW) − 0.71	AV = 0.04, TI = 0.00	0.97
AFDW + TI	52.13	log_10_(ED) = 1.06*log_10_(AFDW) − 0.72	TI = −0.02	0.97
DW + AN	363.2	log_10_(ED) = 1.21*log_10_(DW) − 1.15	AN = 0.14	0.87
DW + AI + AV + TI	364.6	log_10_(ED) = 1.18*log_10_(DW) − 1.10	AI = 0.12, AV = 0.18, TI = 0.17	0.87
DW + AA + TI	364.9	log_10_(ED) = 1.20*log_10_(DW) − 1.14	AA = 0.14, TI = 0.16	0.87
DW + AA	376.5	log_10_(ED) = 1.24*log_10_(DW) − 1.13	AA = 0.09	0.86
DW + AI + AV	377.4	log_10_(ED) = 1.22*log_10_(DW) − 1.10	AI = 0.08, AV = 0.12	0.86
DW + AV + TI	379.8	log_10_(ED) = 1.13*log_10_(DW) − 0.96	AV = 0.10, TI = 0.10	0.86
DW + AV	383.6	log_10_(ED) = 1.17*log_10_(DW) − 1.00	AV = 0.08	0.85
DW + AI + TI	384.1	log_10_(ED) = 1.21*log_10_(DW) − 1.07	AI = 0.06, TI = 0.09	0.85
DW + AI	386.5	log_10_(ED) = 1.23*log_10_(DW) − 1.08	AI = 0.04	0.85
DW + TI	386.7	log_10_(ED) = 1.17*log_10_(DW) − 1.00	TI = 0.07	0.85
DW	387.4	log_10_(ED) = 1.19*log_10_(DW) − 1.02	–	0.85

AICc model selection results for dataset two produced results similar to the previous dataset, with AFDW + AA ranking as the top model in the set (Table 3). Again, the model splitting AA into AI and AV received substantial support and AFDW + AI also received some support. All other models including AFDW as a predictor did not receive empirical support relative to the top model. All models including AFDW were followed in the model selection table by those including DW, then percent protein and lastly, percent lipids (Table 3). It should be noted though that all the models including AFDW or DW as predictors in the reduced dataset had high predictive power with *R* ^2^ > 0.96 (Table 3).

**Table 3:** Regression statistics, log‐likelihood (Log*L*) and Akaike's information criterion corrected for small sample sizes (AICc) for generalized linear models of a trimmed dataset comparing energy density values to percent ash‐free dry weight (AFDW), percent dry weight (DW), percent protein content, and percent lipid content including dummy variables (value of 0 or 1) for large taxonomic groups (AA, aquatic animals; AI, aquatic invertebrates; AV, aquatic vertebrates; aquatic plants and algae were used in the base model and therefore were not given a dummy value). The number of model parameters (*k*), cumulative Akaike weights (*w_i_*), adjusted percent *R* ^2^ values and the difference between the given and best‐fitting model (∆*_i_*) are presented for each candidate model

Model	*k*	Log*L*	AICc	∆*_i_*	*w_i_*	*R* ^2^
AFDW + AA	4	45.66	−81.42	0.00	0.65	0.99
AFDW + AI + AV	5	46.49	−79.97	1.45	0.32	0.99
AFDW + AI	4	42.40	−74.89	6.54	0.02	0.99
AFDW	3	39.07	−71.05	10.37	0.00	0.99
AFDW + AV	4	39.15	−68.40	13.03	0.00	0.99
DW	3	24.44	−41.79	39.63	0.00	0.96
DW + AI	4	24.85	−39.80	41.62	0.00	0.96
DW + AA	4	24.64	−39.37	42.05	0.00	0.96
DW + AV	4	24.60	−39.30	42.12	0.00	0.96
DW + AI + AV	5	24.87	−36.74	44.68	0.00	0.96
Protein + AA	4	−1.02	11.94	93.36	0.00	0.71
Protein + AI + AV	5	−0.88	14.76	96.18	0.00	0.70
Protein	3	−5.74	18.56	99.98	0.00	0.59
Protein + AI	4	−4.73	19.36	100.78	0.00	0.61
Protein + AV	4	−5.71	21.33	102.75	0.00	0.58
Lipid + AI	4	−12.48	34.87	116.29	0.00	0.29
Lipid + AI + AV	5	−12.47	37.95	119.37	0.00	0.26
Lipid + AV	4	−14.32	38.55	119.98	0.00	0.18
Lipid	3	−17.46	42.00	123.43	0.00	0.00
Lipid + AA	4	−17.28	44.47	125.89	0.00	−0.03^a^

a Negative adjusted *R* ^2^ was obtained by fitting a model with low multiple *R* ^2^, using multiple predictors.

**Figure 3:**
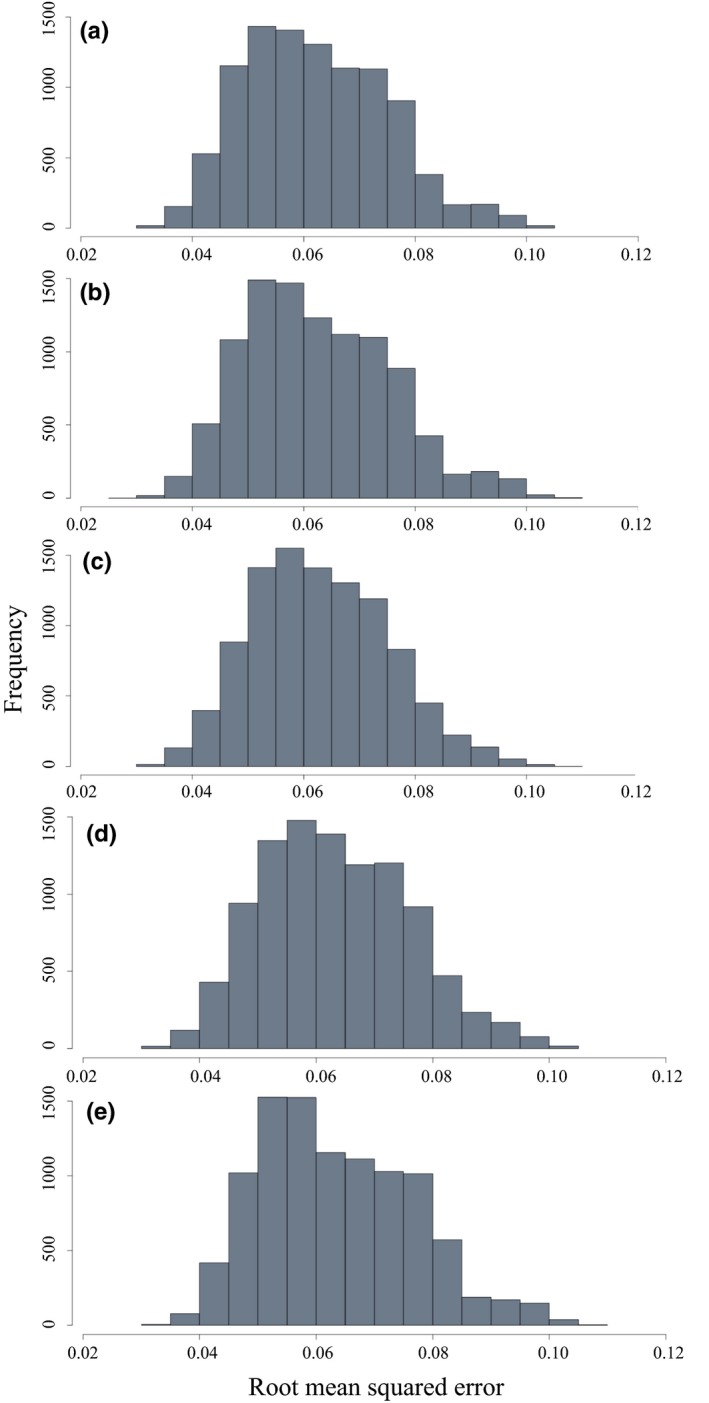
Frequency distribution of root mean squared errors (RMSE) from iterative (*n* = 10,000) cross‐validation between a training (80%) and testing (20%) dataset for the top five best supported models to estimate energy density (ED) from Table 2. (a) ED = AFDW + AA + TI, (b) ED = AFDW + AI + AV + TI, (c) ED = AFDW + AA, (d) ED = AFDW + AI + AV, (e) ED = AFDW + AN

The five best supported models chosen for cross‐validation (Table 2) all included AFDW as a predictor. RMSE values for all models ranged between 0.03 and 0.11 for individual iterations (Figure 3) with an overall mean of 0.06 for all models.

## 4 DISCUSSION

We provide a robust and accurate model to estimate the ED of a taxonomically diverse group of organisms using a simple and easily calculated metric: AFDW. The tight correlation between these two variables (*R* ^2^ = 0.97) represents the association between total organic energy (AFDW) and ED. Organisms of interest need only be weighed for wet and dry weights, then burned in a muffle furnace to obtain ash‐weight (drying and ashing methods described in Cummins & Wuycheck, 1971). Subtracting ash component from the dry weight and dividing this value by the wet weight produces a value for percent AFDW that can then be used to calculate ED using one of the equations listed in Table 2. This procedure takes very little time and has little to no cost requirements besides access to a drying oven and muffle furnace; hundreds of samples can be run in as little as a few days. The prerequisite technical requirements needed to operate a bomb calorimeter or to extract proximate components are also avoided. Weight requirements for the sample are only limited by the power and precision of scales used to measure AFDW. This method can be used to calculate ED for very small organisms and will also allow researchers to easily evaluate individual‐level variability of ED for organisms as small as a few milligrams. The removal of ash‐weight from measurements also eliminates the error associated with indigestible bone, salt, or calcium carbonate fraction can vary considerably among taxa.

AFDW models were improved with the addition of dummy variables for broad taxonomic groups. Correction factors for aquatic animals (AA) and terrestrial invertebrates (TI) resulted in the need for an intercept adjustment of 0.09 and 0.04, respectively. The need for these corrections likely arose from differences between taxonomic groups covered by the dataset. Aquatic plants and algae (APA) were used in the base model to estimate ED when using dummy variables. Differences in ED among taxa for the same AFDW or DW likely arise due to differences in proximate constituents. In particular, APA have structural carbohydrates that make up their cell wall matrices, and thus have typically higher carbohydrate contents than animals (Graham, Graham, & Wilcox, 2009). Low‐ED carbohydrates present in the cell walls of APA would bias the overall model toward lower ED values requiring the appropriate corrections for other taxonomic groups. TI are typically more energy dense than APA (Cummins & Wuycheck, 1971), but can have variable levels of chitin and carbohydrate that contribute to total digestible energy (Bell, 1990). Thus, an AFDW value for TI would produce a higher estimate than expected compared to APA in a model excluding dummy variables, but a lower estimate than expected for AA. By using a model set that includes intercept adjustments (correction factors) for varied taxonomic groups, we provide a robust method for estimating ED across a much wider range of taxa than was available previously.

Our results suggest that AFDW is a superior predictor of ED across taxa compared to previously used metrics: DW, lipid, or protein content (Table 3). Several authors have used constituent predictors to estimate ED of specific groups. Anthony, Roby, and Turco (2000) determined that lipid content was the best determinant of ED variability in fishes. Lipid is roughly twice as energy dense as protein and is easily mobilized for use in metabolic activity, whereas protein is typically more stable, allocated to long‐term musculature associated with growth (Jobling, 1994). Both of these body components are typically translated into ED estimates using conversion factors, but can overestimate values when energetic equivalents are taken from the literature (Craig, Kenley, & Talling, 1978; Schloesser & Fabrizio, 2015). Alternatively, DW is often used as a proxy for ED instead of proximate components due to its ease of use and general applicability that avoids the need to borrow conversion values from the literature. The relationship between DW and ED exists due to the inverse relationship between lipid and water content (Flath & Diana, 1985). Thus, DW is positively related to lipid and protein content as well as ED. Hartman and Brandt (1995) first developed a general multi‐species as well as species‐specific models to estimate ED from DW in fish that have been used in numerous growth and bioenergetics studies (e.g., Johnson & Kitchell, 1996; Penczak, Agostinho, Hahn, Fugi, & Gomes, 1999; Utz & Hartman, 2009). Another general model has been developed specifically for invertebrates using DW (James et al., 2012) that has also been useful in practice (e.g., Deslauriers et al., 2016; Hartman, 2017). To our knowledge, no general model exists to estimate the ED of both invertebrates and vertebrates using AFDW as a predictor. Our results agree with previous findings that suggest DW is a strong predictor of ED (*R* ^2^ = 0.85); however, the removal of ash‐weight from DW estimates greatly improves model performance (*R* ^2^ = 0.97). For some researchers, ash‐weight may not be realistic to acquire if samples need to be retained for other purposes. In these cases, DW models could still provide useful insight (see Table 2 for full equation list of DW models). In instances where taxon‐specific ED models have already been described using DW (e.g., Hartman & Brandt, 1995; James et al., 2012), researchers may not opt to take the extra steps required to measure ash‐weight. Indeed, previously described models relating ED and DW are still appropriate in some situations (e.g., Johnson, Pate, & Hansen, 2017). However, our AFDW model would remain preferable due to its greater accuracy as well as its ability to directly compare aquatic invertebrates, vertebrates, aquatic plants and algae as well as terrestrial invertebrates using a single predictive model.

**Figure 4:**
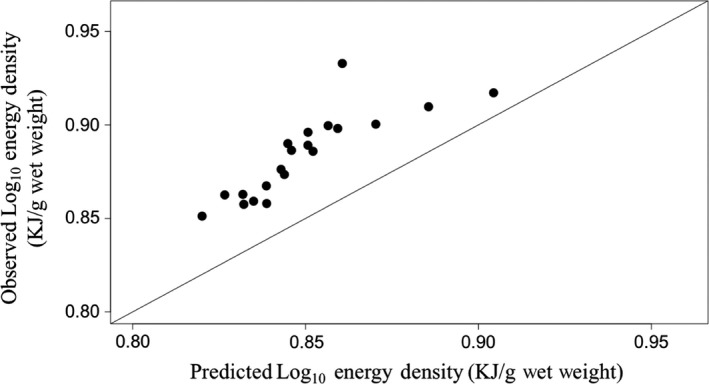
Relationship between observed and predicted values for the energy density of passerine birds investigated in Holmes (1976) and Myrcha and Pinowski (1970). Predicted values estimated using the percent ash‐free dry weight to energy density model including a correction factor for animals as: log_10_(ED) = 1.05*log_10_(AFDW) − 0.78 + 0.08. 1:1 line plotted in Figure

Presently, there is a dearth of data on the ED, DW, and AFDW of terrestrial vertebrates and is limited to passerine birds (Holmes, 1976; Myrcha & Pinowski, 1970). The best‐supported model presented in this paper consistently underestimates the ED values observed in passerine birds by an average of 8.2% (Figure 4). This difference in ED is likely due to fundamental differences in metabolic demands, between terrestrial vertebrates and other organisms examined in our model (Brown, Gillooly, Allen, Savage, & West, 2002). Until further data are available to evaluate the performance of our model on terrestrial vertebrates, we recommend multiplying back‐transformed ED results by a value of 1.08 to account for underestimation in the current model. Still, the relatively close agreement between these data and our model suggests that including terrestrial vertebrates is possible and that further development of this model could provide a general method to estimate the ED of any organism regardless of habitat or taxon.

The application of these models will be of immediate utility in bioenergetics and growth studies that require ED estimates for both consumers and their prey. Regularly, these values are taken from the literature instead of being directly measured or estimated (e.g., Moss et al., 2009; Spitz, Mourocq, Leauté, Quéro, & Ridoux, 2010). Although this practice is commonplace, literature values are often unrepresentative, averaging varied taxa together or borrowing ED values from similar species (Chipps & Wahl, 2008; Ney, 1993). This can introduce substantial error into model estimates. In one example, investigators found that prey consumption was overestimated by as much as 22% when using a model that borrowed ED values from the literature (Johnson et al., 2017). When these values were instead predicted from DW, the authors found a significant reduction in error between observed and predicted values. Analyses like this confirm the accuracy and ease with which these general, multi‐species models can be employed. The benefit of our method over previous estimation models is the generality that it provides across species and systems. For instance, aquatic vertebrates typically feed across multiple trophic levels and can assimilate energy from pelagic, benthic, and terrestrial systems (Pauly, Trites, Capuli, & Christensen, 1998; Vander Zanden & Vadeboncoeur, 2002). The equations provided in Table 2 would be of particular use in these situations where researchers aim to answer questions related to growth, diet, and foraging behavior of complex and interconnected systems.

## CONFLICT OF INTEREST

None declared.

## AUTHOR CONTRIBUTIONS

MT conceived the study and designed the initial data collection strategy; JW collected data, led the data analysis and wrote the original draft of this manuscript; MT, ST, RB, and FJ all contributed significantly to the editing and improvement of earlier drafts and gave final approval for publication.

## Data Availability

All data used to formulate models in this manuscript are freely available from the University of Victoria Research Data Collection (https://doi.org/10.5683/SP2/TZCHKE). Model sources (Chen et al., 2004; Cummins & Wuycheck, 1971; Di Beneditto, Santos, & Vidal, 2009; Doyle, Houghton, McDevitt, Davenport, & Hays, 2007; Dubreuil & Petitgas, 2009; Paine & Vadas, 1969; Pandian & Schumann, 1967; Percy & Fife, 1981; Smirnov, 1962; Thayer, Schaaf, Angelovic, & Lacroix, 1973; Woodland et al., 1968).
